# Understanding the transition from psoriasis to psoriatic arthritis: the role of targeted therapy

**DOI:** 10.1016/j.ero.2025.12.008

**Published:** 2025-12-30

**Authors:** Fadi Kharouf, Matthew Anacleto-Dabarno, Richard J. Cook, Vinod Chandran, Denis Poddubnyy

**Affiliations:** 1 Gladman Krembil Psoriatic Arthritis Program, Centre for Prognosis Studies in the Rheumatic Diseases, Schroeder Arthritis Institute, University Health Network, Toronto, ON, Canada; 2 Division of Rheumatology, Department of Medicine, University of Toronto, Toronto, ON, Canada; 3 Rheumatology Unit, Department of Medicine, Hadassah Medical Center and Faculty of Medicine, Hebrew University of Jerusalem, Jerusalem, Israel; 4 Montreal General Hospital, McGill University Health Center, Montreal, QC, Canada; 5 Department of Statistics and Actuarial Science, University of Waterloo, Waterloo, ON, Canada; 6 Institute of Medical Science, University of Toronto, Toronto, ON, Canada; 7 Department of Laboratory Medicine and Pathobiology, University of Toronto, Toronto, ON, Canada

## Abstract

Most individuals who develop psoriatic arthritis (PsA) first present with psoriasis (PsC), often years before musculoskeletal symptoms emerge. Progression from PsC to PsA is multifactorial, shaped by genetic and environmental influences, and may involve at-risk stages and subclinical stages before culminating in clinically overt arthritis. Consistently identified risk factors include greater PsC severity, nail involvement, arthralgia, and elevated body mass index.

In this Viewpoint, we review and critically appraise current evidence on PsC-to-PsA transition, focusing on how the mechanism of action of biologic disease-modifying antirheumatic drugs may influence this trajectory. Emerging data suggest that therapies targeting the interleukin (IL)-23/IL-17 axis may provide greater protection against PsA development than tumour necrosis factor inhibitors, underscoring the central role of this pathway in psoriatic disease pathogenesis. However, existing studies are limited by confounding, protopathic bias, and heterogeneous cohorts. By integrating mechanistic insights with clinical data, we emphasise the urgent need for rigorously designed multinational randomised controlled trials to determine whether, and how, biologic therapy can modify the natural history of psoriatic disease.

## WHAT IS KNOWN?

Most individuals who develop psoriatic arthritis (PsA) initially experience psoriasis (PsC), often years before joint symptoms emerge [1,2]. The transition from PsC to PsA is a complex and evolving process influenced by both genetic predisposition, such as a family history of the disease, and environmental factors like mechanical stress and infections [3]. This progression typically unfolds through several clinically distinct, though not always sequential, stages [4]. It begins with PsC accompanied by features associated with increased risk of PsA, such as nail disease or obesity, and may advance to a subclinical PsA stage characterised by arthralgia and/or imaging evidence of synovio-entheseal inflammation, ultimately culminating in clinically overt PsA [4].

Several groups have proposed conceptual frameworks to characterise this PsC–PsA transition, using varying terminology and thresholds for preclinical and subclinical disease—among them, the Scher model, the Preventing Arthritis in a Multicenter Psoriasis At-Risk (PAMPA) consensus, and the European Alliance of Associations for Rheumatology task force definitions [[3], [4], [5]]. These frameworks share considerable overlap but differ in how they delineate the boundaries between risk states, subclinical disease, and established PsA, with important implications for both research and clinical practice [[3], [4], [5]].

The median interval from PsC onset to PsA diagnosis is estimated at approximately 7 to 9 years [2,6]; however, transition can occur at virtually any age and at any point during the disease course, including within the first year (concurrent psoriatic disease) or within the first few years after PsC onset [2,4,7,8]. Importantly, there is no evidence of a temporal trend in PsA risk based on PsC disease duration alone [9]. Several clinical features have been consistently associated with an increased likelihood of developing PsA, including greater PsC severity, nail involvement, presence of arthralgia, and elevated body mass index [[10], [11], [12]].

## THE ROLE OF TARGETED DISEASE-MODIFYING ANTIRHEUMATIC DRUGS IN PREVENTING PsA

With the advent of targeted therapies, particularly biologic disease-modifying antirheumatic drugs (bDMARDs) that have shown substantial efficacy in treating PsC, the question has emerged whether early use of these agents can delay or prevent the onset of PsA in patients with PsC [1]. Observational cohort studies, including large-scale registry analyses, have provided early evidence that bDMARDs may modify disease course, with patients receiving bDMARDs generally exhibiting lower rates of PsA development compared to those not receiving such treatments [[13], [14], [15], [16]].

More recent studies have assessed the relative effectiveness of different classes of bDMARDs at reducing PsA risk [[17], [18], [19], [20], [21]]. Several analyses found that interleukin (IL)-23 inhibitors (IL-23i), including the IL-12/23 inhibitor ustekinumab, may offer superior protection against the development of PsA compared to tumour necrosis factor (TNF) inhibitors (TNFi) [[18], [19], [20], [21]]. A relative advantage of IL-17 inhibitors (IL-17i) over TNFi was also found [20,21]. These findings support the hypothesis that the IL-23/IL-17 axis plays a central role in the pathogenesis of musculoskeletal inflammation in psoriatic disease, particularly enthesitis, which is considered an early manifestation of PsA [[22], [23], [24]]. While TNF is proposed to contribute through distinct downstream pathways, the IL-23/IL-17 axis is widely viewed as the predominant driver of disease development [25].

## IMPORTANT LIMITATIONS IN THE CURRENT EVIDENCE

Despite these promising findings, several key limitations and sources of bias must be considered when interpreting the existing literature:

### Confounding by indication and protopathic bias

Confounding by indication poses a major challenge in observational research. For example:

• Patients with more severe PsC are more likely to be treated with IL-17i or IL-23i than with TNFi [1,20].
• Patients with arthralgia, musculoskeletal symptoms, or ultrasound-detected subclinical enthesitis or synovitis, features suggestive of subclinical PsA, may be more likely to be prescribed TNFi [18,26].

Both PsC severity and arthralgia independently increase PsA risk and strongly influence treatment decisions [1,12,20], introducing confounding that may bias observed associations between treatments and PsA development. Although methods such as propensity score matching and inverse probability of treatment weighting can help reduce this bias [18,20,21], unmeasured or residual confounding cannot be fully eliminated.

Additionally, the use of bDMARDs, most commonly TNFi, in patients with ‘undiagnosed PsA’ introduces the risk of protopathic bias [27], a form of reverse causation in which treatment is initiated in response to early manifestations of a disease that has not yet been formally diagnosed, potentially making it appear as though the treatment contributes to disease onset. Consistent with these methodological concerns, Meer et al [28] reported an increased risk of PsA among biologic-treated patients with PsC, an association likely driven by confounding by indication and protopathic bias. When considered alongside studies suggesting a protective effect of IL-23 or IL-17 inhibition [[18], [19], [20], [21],^28^], this finding underscores the inherent limitations of registry-based analyses and reinforces the need for prospective randomised trials.

### Heterogeneity in the composition of cohorts

Variability across cohorts raises important questions about selection bias and the generalizability of findings [29]. For instance, in the study by Gisondi et al [20], the mean PsC duration at bDMARD initiation was 26.7 years, suggesting the cohort included many late transitioners, possibly enriched with HLA-C*06-positive individuals who progress more slowly. The follow-up averaged 4.1 ± 2.1 years, with an annual PsA incidence of approximately 2.4 per 100 person-years [20]. By contrast, the cohort in the study by Strober et al [19] had a shorter mean PsC duration of 1.2 years at baseline, with a follow-up of up to 3 years and an annual PsA incidence of about 5.9 per 100 person-years, despite a similar baseline age (∼47 years). Additionally, 37.4% of patients in the latter study used TNFi, compared to 50.9% in the former [19,20]. Analyses by Singla et al [18], Watad et al [17], and Cho et al [21] also exhibited notable differences in patient characteristics and bDMARD use (Table) [[17], [18], [19], [20], [21]]. Moreover, in some studies [17,20], the relatively small proportions of patients receiving IL-17i or IL-23i limit the strength of conclusions regarding these therapies. Overall, heterogeneity in disease duration and therapeutic approaches in the studied cohorts can explain why findings may not be consistent across studies [29].

**Table:** Selected characteristics of key studies investigating the impact of bDMARD mechanisms of action on progression from PsC to PsA

Study^a^	No. of patients on bDMARDs	Mean age (SD), y	Mean PsC duration (SD), y	Proportion on TNFi (%)	Mean follow-up duration (SD), y	Cumulative PsA annual IR^b^ (95% CI)
Strober et al [19], 2024 (USA)	7345	47.4 (15.5)	1.2 (1.4)	37.4	Up to 3 y	5.9 (5.3-6.5)
Singla et al [18], 2023 (USA)	15,501	50.2 (15.0)	3.3 (3.8)	64.8	2.4 (2.0)	2.6 (NA)
Gisondi et al [20], 2025 (Italy)	622	46.9 (12.9)	26.7 (6.1)	50.9	4.1 (2.1)	2.4 (1.8-3.1)
Watad et al [17], 2024 (Israel)	617	43.3 (15.6)	6.6 (7.5)	58.3	3.5 (2.9)	3.4 (1.9-4.8)
Cho et al [21], 2025 (South Korea)	4522	46.1 (11.5)	NA	25.2	4.9 (3.1)	1.1 (1.0-1.3)^c^

bDMARD, biologic disease-modifying antirheumatic drug; IL, interleukin; IR, incidence rate; NA, not available; PsA, psoriatic arthritis; PsC, psoriasis; TNFi, tumour necrosis factor inhibitor.

Some values were calculated based on data reported in the original articles.

a All studies, except Watad et al [17], reported a reduced risk of PsA development with IL-23 inhibitors, while only Gisondi et al [20] and Cho et al [21] accounted for calendar effects.

b Per 100 patient years.

c A total of 4522 patients were matched using propensity scores, while 1.1 represents the crude incidence rate.

### Low event rates and delayed transition

The relatively low incidence of PsA, combined with the prolonged transition period from PsC to PsA, limits the statistical power of most observational cohorts to identify factors associated with either progression or delayed progression [9,10,12]. A relatively small number of events, particularly in prospective cohorts with relatively short follow-up periods, results in underpowered analyses, wider CIs, and a higher likelihood of type II errors.

### Diagnostic accuracy

With the exception of the study by Gisondi et al [20], which utilised detailed clinical data, most studies relied on registries that, while enabling larger sample sizes, often lack diagnostic precision for PsA due to code-based diagnoses and limited information on imaging findings or classification criteria, potentially compromising data quality [[17], [18], [19],^21^].

### Impact of calendar time

Although all the reported studies were recently published (Table) [[17], [18], [19], [20], [21]], they included patients who started treatment during periods when not all 3 bDMARD classes were available, likely reflecting changes in psoriatic disease management over time. Consequently, calendar time is expected to influence disease outcomes due to both medication availability and secular trends in care. Notably, only the studies by Cho et al [21] and Gisondi et al [20] accounted for this calendar effect, which is an advantage likely to improve the accuracy of their findings.

## WHAT CAN BE DONE?

Despite the mentioned challenges, observational studies provide valuable real-world data that can yield important insights and generate hypotheses. Due to the complexity of the disease, presence of known, poorly measured, and unknown confounding variables, and heterogeneity in risks within and between cohorts, observational data alone cannot definitively answer the key clinical question:

### Do different bDMARDs distinctly affect the risk of transition from PsC to PsA?

To rigorously address this question, a pragmatic randomised controlled trial (RCT) would be required.

In this context, the PAMPA trial represents an important ongoing proof-of-concept study testing whether IL-23 inhibition (guselkumab) can prevent or delay transition from PsC to PsA in a high-risk population [30]. While this trial marks an important step towards preventive intervention [30], its generalizability is limited by its enriched inclusion criteria—moderate-to-severe PsC with imaging abnormalities that, as discussed above, might already indicate an established transition to PsA—as well as by the short duration of blinded treatment (6 months) and the relatively brief follow-up period (2 years) for adequately assessing progression from skin to joint involvement.

Ideally, a definitive prospective interventional study would:

• Enrol patients early in their disease course, preferably before musculoskeletal symptoms develop,
• Randomise participants and stratify them according to established clinical risk factors (eg, HLA status, PsC severity, arthralgia),
• Follow patients long-term with serial imaging assessments.

Enriching a trial population with patients at high risk of progression may enhance feasibility by increasing the frequency of conversion events and enabling more rapid signal detection. A key challenge, however, is that such patients may already represent the earliest stages of PsA rather than a truly preclinical state, necessitating careful case definition and exclusion criteria. Despite these complexities, a large multinational RCT remains the most rigorous means of determining whether bDMARDs can modify the transition from PsC to PsA and of generating the evidence required to guide preventive treatment strategies.

## CONCLUSION

Observational studies provide valuable signals that targeted disease-modifying antirheumatic drugs may impact the risk and progression of PsA in patients with PsC. However, findings vary by study due to confounding by indication, heterogeneity within and between cohorts, protopathic bias, and selection biases. Definitive evidence on how different targeted therapies modify the course of psoriatic disease will require a large, multinational, RCT.

## Competing interests

FK, MA-D, and RJC have no conflicts of interest. VC has received research grants from AbbVie, Eli Lilly, and Johnson and Johnson and has received honoraria for advisory board member roles from AbbVie, Bristol-Myers Squibb, Eli Lilly, Fresenius Kabi, Johnson and Johnson, Novartis, and UCB. His spouse is an employee of AstraZeneca. He serves as a member of the board of directors of GRAPPA and is its co-Vice President. DP has received research support from AbbVie, Eli Lilly, Johnson and Johnson, Novartis, Pfizer, and UCB; consulting fees from AbbVie, Eli Lilly, GlaxoSmithKline, Greywolf Therapeutics, Johnson and Johnson, Merk, Moonlake, Novartis, Pfizer, and UCB; and speaker fees from AbbVie, Canon, Celltrion, Eli Lilly, Globemed, Johnson and Johnson, Medscape, Novartis, Peervoice, Pfizer, and UCB. He serves as a member of the executive committee of ASAS and the steering committee of GRAPPA.
